# Microbial Safety of Beef Along Beef Value Chains in the Ashaiman Municipality of Ghana

**DOI:** 10.3389/fvets.2022.813422

**Published:** 2022-06-22

**Authors:** Vida Yirenkyiwaa Adjei, Gloria Ivy Mensah, Angela Parry-Hanson Kunadu, Kwaku Tano-Debrah, Irene Ayi, Kennedy Kwasi Addo

**Affiliations:** ^1^Department of Bacteriology, Noguchi Memorial Institute for Medical Research, University of Ghana, Accra, Ghana; ^2^Department of Nutrition and Food Science, School of Biological Sciences, University of Ghana, Accra, Ghana; ^3^Department of Parasitology, Noguchi Memorial Institute for Medical Research, University of Ghana, Accra, Ghana

**Keywords:** beef, microbial safety, quality, value chain, Ghana

## Abstract

Food from animal sources continues to be a significant food safety hazard. This study determined the microbial quality and safety of beef along beef value chains with case studies in the Ashaiman Municipality of Ghana. Raw beef samples were collected from four slaughter slabs in the Ashaiman Municipality and analyzed using standard microbiological methods to determine the quality and prevalence of specific pathogens, including *Salmonella* species, *Listeria monocytogenes* (*L. monocytogenes*), and *Brucella* species, as well as *Toxoplasma gondii* (*T. gondii*), *Cyclospora cayetanensis* (*C. cayetanensis*), and *Cryptosporidium parvum* (*C. parvum*). Data regarding food safety knowledge and practices were collected and observed from stakeholders (cattle farmers, butchers, and beef retailers). *Salmonella typhimurium* was isolated from 7.5% (6/80) of the total raw beef samples. However, *L. monocytogenes, Brucella* spp., *T. gondii, C. cayetanensis*, and *C. parvum* were not isolated in this study. The mean level of microbial contamination of beef from the slaughter slabs/abattoir [5.2 Log_10_ colony-forming unit (CFU)/g] was not significantly different (*p* > 0.05) from the mean level observed at retail points (5.4 Log_10_ CFU/g). However, the mean coliform count of 4.3 Log_10_ CFU/g recorded at retail shops exceeded the permissible limits of 10^4^ CFU/g (4 Log_10_ CFU/g) required by the Ghana Standards Authority for safety of meat and carcasses. Knowledge on food safety was at average level for butchers and retailers. Unhygienic practices and poor sanitary conditions at the abattoirs and retail shops observed could be the main contributing factors to microbial contamination of raw beef. Continuous education for meat handlers on issues of food safety and monitoring of slaughter activities will reduce the rate and level of contamination of beef.

## Introduction

Beef is a good source of quality dietary proteins, minerals, and vitamins essential for human metabolic processes (1). Although beef provides essential nourishment for humans, it also provides a rich medium for growth of foodborne pathogens. Beef is the most frequently purchased meat product and constitutes about 52% of meat budget in Ghanaian households (2). It constitutes approximately 27.2% of imported meat products and 17% of domestic meat production (3). Although beef production in Ghana is low, the demand and patronage by consumers are high.

In the informal sector, retailing of beef is carried out in the open under ambient temperatures exposing the beef to flies, bacteria, and other contaminants (4, 5). Consumption of contaminated undercooked meat is the major route of transmission for foodborne infections. Bacterial pathogens, including *Campylobacter, Escherichia coli* (*E. coli*) O157:H7, *Staphylococcus aureus, Salmonella*, and *Enterococci*, are among the top five foodborne pathogens and globally account for 230,000 deaths each year (6). Also, protozoan parasites such as *Toxoplasma gondii* (*T. gondii*) and *Cryptosporidium parvum* (*C. parvum*) are important foodborne pathogens associated with consumption of infected raw or undercooked meat (7, 8). These bacterial and protozoan pathogens are a public health problem and adversely impact the economy in terms of loss of productivity, morbidity, and healthcare cost (9, 10).

Processing of beef along the value chain from slaughter to consumers at retail points is critical due to microbial contamination. Microorganisms may contaminate meat from the hide or intestines of the cattle or from the environmental condition in which animals are reared, slaughtered, transported, and displayed for sale in the markets (11, 12). The mode of transport of meat to the market could also contribute to contamination. It has been observed that meat is commonly transported to the markets in taxis, in head pans, on motor cycles, or on tricycles (13).

With a growing middle class, beef consumption in Ghana is increasing and with an increasing population of an expatriate community in Ghana, restaurants and other food outlets provide alternative processing options such as medium and rare cooked beef that could further increase risk of foodborne illnesses when biological hazards are present. The safety and quality of beef sold to consumers, therefore, needs to be investigated. Cattle farmers, butchers, and retailers who handle meat before it reaches the consumer could play a crucial role in the quality and safety of beef.

Again, in most developing countries such as Ghana where majority of abattoirs/slaughterhouses and meat processing units are substandard and lack modern infrastructure, poorly designed tools and equipment are used. Lack of infrastructure and standards to monitor and control the activities of cattle and beef handlers can easily lead to contamination of beef and beef products and result in food-poisoning incidents, if beef is not cooked thoroughly before consumption. Last, there is much data on the bacteriological quality of beef in Ghana; however, only a few have determined the presence of parasites in meat (14, 15). Therefore, this study aimed to investigate the bacteriological and parasitic quality of beef as well as food safety knowledge and practices of stakeholders in informal beef value chains in the Ashaiman Municipality.

## Materials and Methods

### Study Area and Design

Ashaiman is the capital of the Ashaiman Municipal Assembly, located about 4 km to the North of Tema (industrial city) and about 30 km from Accra, the capital city. It covers a total land area of about 45 km^2^ and falls within latitude 5°42' north and longitude 0°01' west. The municipality has one of the largest cattle markets in the country and plays a central role in the slaughter and sale of beef to other parts of the capital. The vegetation is mainly savannah grasses and shrubs, which provide food for over 27,893 livestock reared by 714 keepers. Ashaiman provides places of residence for most industry workers. However, a large number of persons are involved in cattle, chicken, goat, and sheep rearing. This study was conducted at local abattoirs and slaughter slab in four communities, including old Tulaku, Roman Down, Zenu, and Jericho in the Ashaiman Municipal Assembly. Key information survey preceding exploratory visits and interviews was done with key people at abattoirs to identify the main stakeholders of beef value chains.

### Administration of Questionnaires

A total of 115 stakeholders made up of 25 cattle farmers, 22 butchers, and 68 retailers were conveniently selected and interviewed with structured questionnaire. The butchers and retailers' questionnaire captured information on demographics, acquisition of slaughter cattle, transport of beef to the retail outlets and the markets, and handling and storage of beef. Cattle farmers were interviewed with structured questionnaire to solicit information on the sources of animals, farm practices, transport distance, animal handling condition during transport, location, and training. The last section, which consisted of 6 questions, tested their knowledge on foodborne disease (FBD) and food safety. Scoring of food safety knowledge was done using scoring method described by Nee and Sani (16). Respondents were asked to choose from three options—yes, no, or do not know. The terms “correct” and “wrong” were used to indicate correct and wrong answers, respectively, by the respondents. The score ranged between 0 and 6, which was converted to 100 points and expressed as percentages. The score below 50% was defined as poor knowledge, while score above 70% was regarded as good knowledge. A checklist on items and facilities required for good hygienic practices in the handling of raw beef by butchers and retailers was used for audit.

### Microbiological Analysis

#### Sample Collection

A total of 80 raw beef samples were collected aseptically from four (4) slaughter slabs and 12 butcher shops/retail outlet traced from the identified abattoir/slaughter slabs. Out of these, 20 samples were obtained from the four slaughter slabs, while 60 beef samples were collected from 12 different retailers located in the Ashaiman market. On each sampling day, 16 raw beef samples, each weighing 100 g, were purchased and collected aseptically into sterile polythene pouches and sealed and transported on ice to the Bacteriology Laboratory at the Noguchi Memorial Institute for Medical Research. Sample collection was repeated for 5 weeks.

#### Total Plate Count and Total Coliform Count

A total of 10 g of beef sample was aseptically homogenized in 90 ml phosphate-buffered saline (PBS). 1 ml of the homogenate was diluted in 9 ml PBS tubes to obtain 10^−1^ dilution factor. Serial dilutions up to 10^−4^ were prepared for the colony count. Aliquot of 1 ml of each of serial dilution was transferred to two (2) petri dishes (4-inch diameter) labeled plate count agar (PCA) and MacConkey agar (MAC) each and molten plate count agar (PCA) and MacConkey agar (MAC) (15–20 ml) were poured on them, respectively. Plates were gently swirled to uniformly mix the sample. The plates were inverted and incubated at 37°C for 24 h. The incubated plates were examined for bacterial colonies and were counted using a colony counter.

#### Isolation and Identification of *Brucella* spp.

A loopful (0.1 ml) of the stock homogenate was streaked on selective *Brucella* agar, prepared aseptically by following the manufacturer's instructions using *Brucella* Medium Base (CM0169), *Brucella* Selective Supplement (SR0083), and 5.00% inactivated horse serum all from Oxoid, Basingstoke, UK. The plates were incubated at 35°C in a humidified incubator with 5 to 10% CO_2_ for 72 h. After incubation, punctate colonies that were nonpigmented and nonhemolytic were regarded as presumptive. The presumptive *Brucella spp*. were streaked on nutrient agar and incubated at 37°C for 24 h. Purified colonies from the nutrient agar plates were confirmed according to procedures described by Alton et al. (17). Pure colonies of presumptive *Brucella* spp. were Gram stained and confirmed by panel of test such as oxidase production, CO_2_ dependence, catalase production, and urea production.

#### Isolation and Identification of *Salmonella* spp.

Isolation and identification of *Salmonella* spp. were performed using procedures previously described by Addo et al. (18). 1 ml aliquots stock homogenate prepared earlier were transferred into 10 ml Rappaport–Vassiliadis Broth for enrichment. Samples in Rappaport–Vassiliadis Broth (Oxoid, CM0669) were incubated at 37°C for 24 h. 0.1 ml of the enriched samples were then streaked onto *Salmonella–Shigella* Agar (SSA) (Oxoid, CM0099) and incubated at 37°C for 24 h. Cream colonies with black centers on the SSA presumed to be *Salmonella* spp. were purified on nutrient agar and confirmed using Gram staining, analytical profile index (API) (20E, Biomérieux, France), and *Salmonella* latex agglutination.

#### Isolation of *Listeria monocytogenes*

A total of 10 g of raw beef was homogenized in 9 ml half Fraser Broth (Oxoid, CM0895) and incubated at 30°C for 24 h to obtain a primary enrichment broth. 0.1 ml of the primary enrichment broth was introduced in 10 ml of Fraser Broth (Oxoid, CM0895) and incubated at 37°C for 48 h (secondary enrichment broth). Both the primary and secondary enrichment broths were subcultured on *Listeria* chromogenic agar plates (Oxoid, CM1084) and incubated aerobically at 37°C for 48 h. Colonies appear blue-green with opaque halos, presumptive of *Listeria* spp. that were purified and confirmed. Catalase test was done to confirm *Listeria monocytogenes (L. monocytogenes*) by smearing pure colonies on a clean glass slide with a sterile inoculating loop. Three drops of 3% hydrogen peroxide were placed on the smear and the slide was observed for bubbles. Colonies on the blood agar plates were observed for hemolysis to confirm *L. monocytogenes*.

### Detection of *Toxoplasma gondii* by PCR

#### Deoxyribonucleic Acid Extraction From Beef Tissue

Beef samples were minced and 25 mg of each minced tissue was used for DNA extraction following the manufacturer's instructions of a commercial DNA extraction kit (DNeasy^®^ Blood and Tissue Kit, Qiagen, USA). All the extracted DNA samples were stored at −20°C until used.

#### Nested PCR Amplification

The extracted DNA was analyzed by a nested PCR (nPCR) method using the appropriate primer sets in a method employed by Prestrud et al. (19) with modification. Nested one PCR mixture contained 1X PCR buffer, 2.5 mM MgCl_2_, 2.0 mM each of dNTPs, 0.1 μM each of forward and reverse primers, 0.5 units of Taq polymerase, and 5 μl of DNA extract. The nested one reaction condition was set and maintained at 95°C for 4 min, followed by 25 cycles of 94°C for 30 s, 55°C for 1 min, and 72°C for 1.5 min. For nested two reaction, the mixture contained 1X PCR buffer, 2.5 mM MgCl_2_, 2.0 mM each of dNTPs, 0.3 μM each of forward and reverse primers, 0.5 units of Taq polymerase, and 1 μl of nested one amplicons. The nested 2 reaction condition was maintained at 95°C for 4 min, followed by 35 cycles of 94°C for 30 s, 60°C for 1 min, and 72°C for 1.5 min. 7 μl of each the nested PCR product was loaded into a 2% agarose gel and ran for 1 h at 80 V. The gel was viewed under UV in a transilluminator to identify any bands corresponding to *T. gondii* (225 bp for *SAG3* gene and 344 bp for *GRA6* gene).

#### Detection of *Cryptosporidium parvum* by ELISA

There is very little published data on elution of *Cryptosporidium* oocyst from meat products. Therefore, the procedures used in this study were adopted and modified from previous studies of Robertson and Huang (20) who eluded oocyst from cured meat. Elution of *Cryptosporidium* oocyst was performed by homogenizing 10 g of raw beef in a stomacher bag containing 90 ml normal saline and Tween-20 for 15 min. The supernatant of the homogenate was aliquoted into clean vials and stored at −80°C until analysis. Aliquoted samples were thawed to room temperature before use. *Cryptosporidium* assay was performed as described by Jafari et al. (21). Ag-ELISA Kit (Cypress Diagnostics, Belgium) was used and the manufacturer's instructions were followed. Although this method was used to detect *Cryptosporidium* oocyst in stool, it was adopted in this study due to its high sensitivity (100%) and specificity compared to acid-fast staining and co-agglutination (21–23). 50 μl of sample, positive and negative controls was added to the ELISA plate. 50 μl of enzyme conjugate reagent was added immediately and covered using adhesive plastic. The mixture was incubated for 60 min at room temperature. Following incubation, the plate was washed four times with washing buffer reagent. 100 μl of chromogen/substrate reagent was added to each well and incubated in a dark room for 15 min. Then, 50 μl stop solution was added. Reaction optical density was read at 450 nm in <15 min using absorbance-based microplate reader. A positive reaction was calculated to be double the optical density value of the negative control.

#### Detection of *Cyclospora cayetanensis* by Modified Acid-Fast (Modified Ziehl–Neelsen) Staining

A total of 20 g of beef was homogenized in 50 ml phosphate-buffered saline Tween-20 (PBST) for 30 s. The homogenate was filtered through a 3.0-μm cellulose nitrate membrane pore after which the cellulose membrane was suspended in 10 ml PBST, vortexed for 60 s, and centrifuged for 15 min at 3,000 rpm. The pellets obtained were used to prepare a smear and stained and observed microscopically to detect *Cyclospora cayetanensis* (*C. cayetanensis*) oocyst, which appear light pink to dark purple and measures about 8 to 10 μm.

### Statistical Analysis

The data obtained from the microbiological examination of the carcasses were analyzed using SPPS version 20 (IBM Incorporation). The counts were expressed in log colony forming units per gram of sample [Log_10_ colony-forming unit (CFU)/g]. One–way ANOVA was used to determine the statistical significance (*P* < 0.05) of the total plate count (TPC) and total coliform count (TCC) at the slaughter slabs and retail outlets. The presence of pathogens was presented as percentages, while results of survey and audits are given in Tables.

## Results

The results in Table 1 showed the demographic characteristics of stakeholders (cattle farmers, butchers, and retailers). All the 115 interviewed were males, mostly Muslims and 40% in the 30–39 years' age range. About 40.9% had no formal education. However, vocational school was the highest level of education attained by those who had been to school. Majority (74.3%) of the stakeholders had not been trained in animal hygiene (for cattle farmers) or meat hygiene. The overall food safety knowledge score (level) was average for both the butchers (59.1%) and retailers (57.6%) (Table 2). However, audits result in Table 3 showed that they do not always put the knowledge into practice. It was observed that only 16.2% of retailers had handwashing basin in service area and preparation area, 23.5% of retailers wore apron or head gear, and 29% of retailers used nets or glass as screens to protect meat from flies and dust.

**Table 1:** Demographic characteristics of farmers, butchers and retailers along beef value chain in Ashaiman Municipal area.

**Characteristic**		**Farmers (*N* = 25) *n* (%)**	**Butchers (*N* = 22) *n* (%)**	**Retailers (*N* = 68) *n* (%)**
Age group (years)	<20	0 (0)	0 (0)	0 (0)
	20–29	1 (4)	6 (27.2)	13 (19.1)
	30–39	7 (28)	11(50)	28 (41.2)
	40–49	14 (56)	5 (22.7)	22 (32.4)
	>50	3 (12)	0 (0)	5 (7.4)
Gender	Male	25 (100)	22 (100)	68 (100)
Education	No formal	10 (40)	10 (45.5)	27 (39.7)
	Basic school	10 (40)	9 (40.9)	32 (47.1)
	SHS/vocational	5 (20)	3 (13.6)	9 (13.2)
Religion	Christian	1 (4)	0 (0)	0 (0)
	Muslim	24 (96)	22 (100)	68 (100)
Years in business	1–5	0 (0)	5 (22)	20 (29.4)
	6–10	1(4)	9(40.9)	20 (29.4)
	11–15	4 (16)	4 (18.1)	9 (13.2)
	16–20	10 (40)	3 (13.6)	11(16.2)
	>20	10 (40)	1 (2.5)	8 (11.7)

*Total Number of Participants = 115*.

*SHS, Senior High School*.

**Table 2:** Knowledge level score of Butchers and Retailers on Foodborne disease and food safety.

**Characteristics**		**Butchers (*N* = 22) *n* (%)**	**Retailers (*N* = 68) *n* (%)**
Have you heard of FBD	Yes	10 (45.5)	59 (86.8)
	No	10 (45.5)	0 (0)
	Don't know	2 (9.0)	9 (13.2)
Can you give examples of FBD	Correct	10 (45.5)	22 (32.4)
	Wrong	0 (0)	0 (0)
	Don't know	12 (54.5)	46 (67.6)
Can beef consumption cause FBD	Yes	20 (90.9)	28 (41.2)
	No	0 (0)	29 (42.6)
	Don't know	2 (9.0)	11 (16.2)
What are some of the symptoms of FBD	Correct	14 (63.6)	51 (75)
	Wrong	0 (0)	0 (0)
	Don't know	8 (36.4)	17 (25)
How can you prevent FBD	Correct	18 (81.8)	32 (47.0)
	Wrong	0 (0)	31 (45.6)
	Don't know	4 (18.2)	5 (7.4)
What do you do to ensure that the beef you sell will not cause FBD?	Correct	6 (27.3)	43 (63.2)
	Wrong	16 (72.7)	25 (36.8)
Have had formal training course on food safety?	Yes	6 (27.3)	18 (26.5)
	No	16 (72.7)	50 (73.5)
**Mean knowledge score**		**59.10%**	**57.60%**

**Table 3:** Hygienic practices of butchers/retailers in Ashaiman municipality.

**Facility/Practices**	**Percentage of butchers/retailers (%)**
Hand wash basin in service area	16.2
Sink with running water for hand washing	0
Hand wash basin in back preparation area	5.8
Availability of soap for washing hands	61.7
Towels for drying hands	11.7
Other sanitary facilities (local detergent)	29.7
Use of aprons and/or head cover	23.5
Use of screen to protect meat from flies	29.4

All the 25 cattle farmers practiced the extensive system of raising cattle. Animals were bred by the farmer or purchased from nearby farms or other parts of the country such as Techiman, Yepi, and Tamale and neighboring countries such as Burkina Faso, Niger, and Mali. All the butchers (100%) dressed their carcasses by singeing with car tires or firewood. Cleaning, evisceration, and cutting of carcass were done on concrete slabs, but hanged for inspection. However, beef was not chilled after evisceration and after cutting prior to transportation to the markets and retail points. The most popular means of transport of meat to retail shop in this study was taxi (64.7%) (Table 4). Carcasses were packaged on polyethylene or cardboards in vehicles for transport. Averagely, it took butchers/retailers <1 h to transport meat to their retail shops. None (100%) of the butchers transported meat in refrigerated meat vans or on ice.

**Table 4:** Transportation of beef to retail shops.

**Item**	**Number of butchers and retailers (%) *n* = 68**
**Means of transport**	
Taxi	44 (64.7)
Mini truck	17 (25)
Motor bike	7 (10.3)
**Temperature during transport**	
On ice	0 (0)
Without ice	68 (100)
Refrigerated meat van	0 (0)

The mean TPC of beef at the slaughter slabs and retail outlets was 5.2 and 5.4 Log_10_ CFU/g, respectively, while the mean TCC of beef at the slaughter slabs and retail outlets was 3.7 and 4.3 Log_10_ CFU/g, respectively (Table 5). There was no significant difference between the TPC and TCC at the slaughter slabs and retail outlets (*P* = 0.58). *Salmonella typhimurium* (*S. typhimurium*) was detected in six (7.5%) of the total beef samples (Table 5). *L. monocytogenes, Brucella* spp., *T. gondii, C. parvum*, and *C. cayetanensis* were not detected in this study. However, other bacterial species were isolated with *E. coli* being predominant (29%) (Table 6).

**Table 5:** Mean microbial count [in Log_10_ (CFU)/g] at slaughter slabs/abattoirs and detection of specific pathogens.

	**Slaughter slab**			**Retail outlet**		
**Location**	**TPC**	**TCC**	***Salmonella* Typhimurium**	**TPC**	**TCC**	***Salmonella* Typhimurium**
Tulaku	4.7^a^	4.1^a^	-	5.5	4.4	++
Zenu	5.3^b^	3.8^b^	-	5.3	4.6	+
Roman down	5.6^b^	3.3^b^	-	5.5	3.8	-
Jericho	5.2^b^	3.6^b^	+	5.3	4.5	++
**Overall mean**	**5.2**	**3.7**		**5.4**	**4.3**	

*Means in the same column with different superscript are significantly different*.

*TPC, Total plate count; TCC, Total coliform count*.

*+, Detected in one sample; ++, detected in two samples; -, Not detected*.

**Table 6:** Frequency and percentage of pathogens and other bacterial species from 80 raw beef samples obtained from slaughter slabs and retail shops.

**Pathogen**	**Total no. (%)**	**Slaughter slab**	**Retail**
*L.monocytogenes*	0 (0)	0	0
*Brucella spp*.	0 (0)	0	0
*T. gondii*	0 (0)	0	0
*C. parvum*	0 (0)	0	0
*C. cayetanensis*	0 (0)	0	0
*E. coli*	19 (29)	9	10
*S. aureus*	3 (5)	1	2
*K. pneumoniae*	7 (11)	2	5
*Streptococcus* spp.	7 (11)	0	7
*Citrobacter* spp.	4 (6)	0	4
*P. aeruginosa*	3 (5)	1	2
*Proteus* spp.	6 (9)	1	5
*Bacillus spp*.	7 (11)	2	5
*Enterococcus faecalis*	4 (6)	0	4
*Enterobacter cloacae*	5 (8)	0	5
**Total**	**65**	**16**	**49**

## Discussion

Butchering and sale of meat at Ashaiman Municipality were mostly done by young middle-aged Muslim men within 20–49 years' age range, which is similar to findings of Frimpong et al. (13) in Kumasi. Butchering is a profession, which requires much energy and physical strength to travel several times in a week to purchase livestock from livestock market and restrain animals for slaughter (24). It is not surprising that about 40% of the study participants in this study were young males. Education and training of meat handlers about the basic concept of meat hygiene and good manufacturing practices are important in safeguarding the quality and safety of meat to consumers. This study showed that 59.1% of the participants have had some form of formal education and 25% of the participants had training on hygiene or food safety. Bhandare et al. (25) reported that abattoir workers in most developing countries are untrained and, thus, pay no attention to hygienic practices and, therefore, contribute to bacterial contamination.

Total plate count used to measure the general bacterial load to reflect the level of contamination is a useful tool in monitoring meat quality. For beef to be considered unwholesome, the TPC should exceed 7 Log_10_ CFU/g, which is the International Commission on Microbiological Specification of Food (ICMSF) (26). By the Ghana Standards Authority (GSA) criteria, the TPC should not exceed 10^6^ CFU/g (6 Log CFU/g). In this study, the mean TPC at the slaughter slab/abattoir and retail shops was 5.2 and 5.4 Log_10_ CFU/g, respectively, and, thus, within the range of permissible limit of both the GSA and the ICMSF. This finding is comparable to those reported by Ahmad et al. (27) in Pakistan and Anachinaba et al. (14) in Ghana, who recorded counts ranging from 4.33 to 6.7 Log_10_ CFU/g. The microbial count enumerated from fresh raw beef indicated that the beef samples were contaminated. The possible source of contamination may include the processing area, knives, gut content, hide, meat handlers, vehicle for transporting carcass, and selling environment. It must be noted that in this study, samples were collected early in the morning, which are actually expected to be of the best quality, as the beef is freshly processed. The results also highlight the level of hygiene with respect to beef handling and storage at the retail shops. The production chain in all the slaughter slabs was poorly organized. Cleaning of carcass after singeing to cutting of meat for inspection was all done on the bare floors that were stained with blood and gut content from previous slaughter. Though the TPC of beef was within the limit considered as wholesome for consumption, the presence of pathogens such as *E. coli* and *Salmonella*, which are known to cause foodborne infections, is of public health concern (4). The mean total coliform count (TCC) recorded at the slaughter slabs and retail shops in this study was 3.7 and 4.3 Log_10_ CFU/g, lower than counts reported by Twum (5) in Ghana, which ranged from 5.29 to 5.48 Log_10_ CFU/g. In Nigeria, however, Adetunji et al. (28) reported high TCC in beef with ranges from 0 to 8.21 Log_10_ CFU/g. High TCC recorded in this study, which exceeded the permissible limits of 3 Log_10_ CFU/g required by the GSA (2013) and the ICMSF (26), suggests that the beef samples were of poor quality. The presence of coliforms in meat is an indication of poor processing activity, which was done mainly on contaminated abattoir floors and lack of separation between dirty and clean area in this study. Contamination of the beef with fecal matter could have been from the environment, flies, and other materials, including contaminated water. The 7.5% prevalence of *Salmonella* reported in this study was low compared to the 31% reported by Adzitey (29) who determined the prevalence of *Salmonella* spp. and *E. coli* in beef samples sold at Tamale Metropolis in Ghana. The low prevalence of *Salmonella* spp. recorded in this study is similar to previous studies (5, 18, 30). Isolation of *S*. typhimurium indicates a public health concern and may pose a health hazard, if beef is eaten undercooked or cross-contamination occurs during food preparation (31). The presence of *Salmonella* spp. in the meat samples is also an indication of poor hygienic practices during processing from the farm to the retail shops.

*Listeria monocytogenes* was not isolated in this study, though it was reported to be the etiological agent for FBD outbreak in South Africa, which claimed 180 lives (32). Manifestations of listeriosis include meningitis and spontaneous abortion or stillbirth in pregnant women. The ability of *L. monocytogenes* to multiply in various foods at temperatures as low as 2 to 4°C makes the occurrence of *L. monocytogenes* in food products, of particular concern (33). The prevalence of *L. monocytogenes* and *Brucella* spp. in meat, though not found in this study, is also an indication of unhygienic meat processing (30). *T. gondii* was not detected in beef in this study, which agrees with previously published report (34). Low prevalence of 1.7 and 4% has been reported from similar studies by Rahdar et al. (8) and Hosein et al. (35) in United Kingdom (UK) and Iran, respectively. The absence of *T. gondii* in beef from this study could be that the cattle were not exposed to the infective oocyst probably due to low cat population, which are the definitive host. The findings could also confirm that cattle are able to clear the oocyst after ingestion and are, thus, resistant to the infection (36). Neither *C. parvum* nor *C. cayetanensis* was isolated from any of the beef samples in this study, which corroborates data by Eberhard et al. (37). *C. parvum* and *C. cayetanensis* are emerging foodborne pathogens shed through the feces of chicken and dogs. However, cattle are not known to be colonized by *Cyclospora* spp. (38). Rather, irrigation water used for production of crops usually eaten raw has shown widespread presence of these parasites (39). The absence of these protozoan parasite in this study supports suggestions that cattle show lower susceptibility to these protozoan infections. Another possible explanation could be that the meat of the cattle that were sampled for this study may have not been exposed to the parasites.

Though the food safety knowledge of both the retailers and butchers was average in this study, 74.3% had not been trained on food safety and/or meat hygiene. Of those trained, 25.7% had only one training organized by the Ministry of Food and Agriculture. Some slaughter slabs/abattoirs had veterinary officers and meat inspectors at post to do antemortem and postmortem inspection of cattle and beef, respectively, before distribution to the retail shops. Although the total plate count was comparable for the farm and retail outlets, the coliform count was much higher for the samples from the retail outlets. This indicates that a transdisciplinary approach is required because merely ensuring that the quality of meat at the abattoir level is good due to the presence of trained meat inspectors and veterinarians, without ensuring that all the stakeholders in the value chain work in synergy to ensure that meat remains wholesome before consumption will not achieve desired outcomes. Value chain analysis is an essential starting point for a One Health approach to meat safety. A great example was set about a decade ago, during the avian influenza pandemic when the One Health approach was used to bring together several players from public and animal health disciplines to manage the pandemic (40). These interdisciplinary efforts mobilized value analysis as a tool to map actors, processes, and value creation to plan disease control and assess the impact of the disease and control measures (41). For beef safety, within the context of Ghana, collaborative efforts, involving veterinarians, herdsmen, butchers, public health experts, physicians and other related professionals, food and environmental regulatory authorities, district assemblies, and consumers, must work together for control and prevention of zoonotic infection transmission between the human–animal interface. There is a need to educate and train cattle farmers and beef/meat handlers to improve in sanitation and hygiene to reduce microbial contamination of beef and transmission of zoonotic pathogens to humans and the environment. Continuous surveillance by the regulatory authorities and insistence on the establishment of hazard analysis and critical control points (HACCPs) by meat retail outlets would ensure that the consumer is protected from unwholesome meat. Of course, while laws ensure compliance, a participatory approach will be critical to the success of any transdisciplinary approach. Hence, all the identified stakeholders in the beef value chain must be given the opportunity to participate in the process, so that there is a sense of ownership that will lead to sustainably any efforts at improving beef quality and safety.

## Conclusion

Food safety in the beef production chain requires training and collaboration with all the partners and veterinarians play an important role. This study revealed that beef sold in the municipality is contaminated with pathogens such as *S. typhimurium, E. coli*, and *Staphylococcus aureus* (*S. aureus*). Though the prevalence of *S. typhimurium* in this study was lower (7.5%) than that described in previous studies from Ghana, its presence together with other pathogens isolated is a public health concern. *L. monocytogenes* and *Brucella* spp. were not isolated in any of the beef samples. The absence of these pathogens is good, but consumers of beef need to be aware that meat should be cooked thoroughly at temperatures above 75°C in order to kill all pathogens, which may be present in beef. This study makes a case for using a One Health approach to achieve food safety.

## Data Availability Statement

The data analyzed in this study is subject to licenses/restrictions. Requests to access these datasets should be directed to vyadjei@gmail.com.

## Author Contributions

VA, KA, AP-HK, and KT-D designed the study. VA wrote the protocol and first draft, did the sample collection, and performed the bacterial isolation and identification. IA provided technical support for parasite identification in the Parasitology Department of NMIMR. GM and VA performed the data analysis. GM, VA, KA, IA, AP-HK, and KT-D reviewed the manuscript. All authors have read and approved the final version of the manuscript.

## Funding

This study was funded by the DELTAS Africa Initiative (Afrique One—ASPIRE/DEL-15-008). Afrique One—ASPIRE was funded by a consortium of donors, including the African Academy of Sciences (AAS), Alliance for Accelerating Excellence in Science in Africa (AESA), the New Partnership for Africa's Development Planning and Coordinating (NEPAD) Agency, the Wellcome Trust (107753/A/15/Z), and the UK government.

## Conflict of Interest

The authors declare that the research was conducted in the absence of any commercial or financial relationships that could be construed as a potential conflict of interest.

## Publisher's Note

All claims expressed in this article are solely those of the authors and do not necessarily represent those of their affiliated organizations, or those of the publisher, the editors and the reviewers. Any product that may be evaluated in this article, or claim that may be made by its manufacturer, is not guaranteed or endorsed by the publisher.

## References

[1] Cabrera M Cabrera MC Saadoun A. An overview of the nutritional value of beef and lamb meat from South America. MESC. (2018) 98:435–44. 10.1016/j.meatsci.2014.06.033 25042240

[2] Osei-Asare YB Eghan M. Meat consumption in ghana, evidence from household micro-data. Empir Econ. (2014) 13:8997.

[3] MoFA. Medium Term Agricultural Sector Investment Plan (METASIP) II, 2014 – 2017. Accra Ghana: Ministry of Food and Agriculture. (2017).

[4] Soyiri I Agbogli H Dongdem J. A pilot microbial assessment of beef sold in the Ashaiman market, a Suburbof Accra, Ghana. Af J Food Agric Dev. (2008) 8:91–103. 10.4314/ajfand.v8i1.19182

[5] Twum E,. Microbial quality of fresh beef sold in the Birim north district of the Eastern Region of Ghana (dissertation/master's thesis). Kwame Nkrumah University of Science Technology, Kumasi, Ghana (2015). Available online at: https://docplayer.net/94536828-Kwame-nkrumah-university-of-science-and-technology-kumasi-college-of-science-microbial-quality-of-fresh-beef-sold-in-the-birim-north-district.html

[6] WHO. WHO estimate the global burden of foodborne Diseases. (2015). Available online at: http://www.who.int/foodsafety/publications/foodborne_disease/fergreport/en/re (assessed May 4, 2017).

[7] Boughattas S Ayari K Sa T Aoun K Bouratbine A. Survey of the Parasite *Toxoplasma gondii* in human consumed ovine meat in tunis city. PLoS ONE. (2014) 9:e85044. 10.1371/journal.pone.0085044 24427300 PMC3888417

[8] Rahdar M Samarbaf-Zadeh AR Arab L. Evaluating the Prevalence of Toxoplasma gondii in Meat and Meat Products in Ahvaz by PCR Method. Jundishapur J Microbiol. (2012) 4:570–3. 10.5812/jjm.4280

[9] Pal M Ayele Y Jadhav VJ. Epidemiology public health importance of foodborne protozoan diseases. Indian J Vet Pub Health. (2017) 3:7–12. Available online at: https://www.researchgate.net/publication/318452050

[10] Scallan E Griffin PM Angulo FJ Tauxe RV Hoekstra RM. Foodborne illness acquired in the United States-unspecified agents. Emerg Infect Dis. (2011) 17:16–22. 10.3201/eid1701.P21101 21192849 PMC3204615

[11] Obeng AK Johnson FS Appenteng SO. Microbial quality of fresh meat from retail outlets in tolon and kumbungu districts of the northern region of Ghana. Int J Sci Technol. (2013) 2:423–8.

[12] Koffi-Nevry R Koussemon M Coulibaly SO. Bacteriological quality of beef offered for retail sale in Cote d'ivoire. Am J Food Technol. (2011) 6:835–42. 10.3923/ajft.2011.835.842

[13] Frimpong S Gebresenbet G Bosona T Bobobee E Aklaku E Hamdu I. animal supply and logistics activities of abattoir chain in developing countries : the case of Kumasi Abattoir, Ghana. J Servi Sci Mgt. (2012) 5:20–7. 10.4236/jssm.2012.51003

[14] Anachinaba IA Adzitey F Teye GA. Assessment of the microbial quality of locally produced meat (Beef and Pork) in Bolgatanga Municipal of Ghana. J Food Safety17. (2015) 7:1–5. Available online at: http://www.udspace.uds.edu.gh/handle/123456789/584

[15] Hughes FA Adu-gyamfi A Appiah V. Microbiological and parasitological quality of local beef retailed in accra and radiation sensitivity of salmonella spp. Int. J. Curr. Microbiol. App. Sci. (2015) 4:86–96. Available online at: https://www.academia.edu/13716769/

[16] Nee SO Sani NA. Assessment of Knowledge, Attitudes and Practices (KAP) Among food handlers at residential colleges and canteen regarding food safety. Sains Malays. (2011) 40:403–10.

[17] Alton GG Jones LM Angus RD Verger JM. Techniques for the brucellosis laboratory. In: Institut National de la recherche Agronomique. Paris: INRA Publications (1988). p. 190-2.

[18] Addo K Adjei V Mensah G Jackson-Sillah D. Microbial quality and antibiotic residues in raw beef from selected abattoirs in Accra, Ghana. Int J Trop Dis Health. (2015) 6:20–6. 10.9734/IJTDH/2015/14759

[19] Prestrud KW Asbakk K Fuglei E Mork T Tryland M. Direct high-resolution genotyping of Toxoplasma gondii in arctic foxes (Vulpes lagopus) in the remote arctic Svalbard archipelagoreveals widespread clonal Type II lineage. J. Vet. Parasitol. (2008) 158:121–8 10.1016/j.vetpar.2008.08.020 18922642

[20] Robertson LJ Huang Q. Analysis of cured meat products for cryptosporidium oocysts following possible contamination during an extensive waterborne outbreak of cryptosporidiosis. J Food Prod. (2012) 75:982–8. 10.4315/0362-028X.JFP-11-525 22564952

[21] Jafari R Maghsood AH Safari M Latifi M. Comparison of fecal antigen detection using enzyme linked immunosorbent assay with the auramine phenol staining method for diagnosis of human cryptosporidiosis. Jundishapur J.Microbiol. (2015) 8:1–5. 10.5812/jjm.16470 25825642 PMC4376972

[22] Al-megrin WAI. Comparison of ELISA and microscopy for detection of *cryptosporidium* oocyst in animals. Pakistan J Biol Sci. (2015) 18:341–5. 10.3923/pjbs.2015.341.345

[23] Michel M Khalifa A Ibrahim I. Detection of cryptosporidium parvum antigen by co-agglutination test and ELISA. Eastern mediterr. Health J. (2000) 6:898–907. 10.26719/2000.6.5-6.898 12197347

[24] Adzitey F. Effect of pre-slaughter animal handling on carcass and meat quality: Mini review. Int Food Res J. (2011) 18:485–91. Available online at: http://www.ifrj.upm.edu.my/IFRJ-2010-140.pdf

[25] Bhandare SG Sherikarv AT Paturkar AM Waskar VS Zende RJ. A comparison of microbial contamination on sheep/goat carcasses in a modern Indian abattoir and traditional meat shops. Food Control. (2007) 18:854–68. 10.1016/j.foodcont.2006.04.012

[26] ICMSF. Microorganisms in Foods. 5th ed. Gaithersburg: Aspen Publishers, Inc. (1996).

[27] Ahmad MUD Sarwar A Najeeb MI Nawaz M Anjum AA Ali AA. Assessment of microbial load of raw meat at abattoirs and retail outlets. J Anim Plant Sci. (2013) 23:745–8. Available online at: https://www.researchgate.net/publication/287524023

[28] Adetunji VO Adesokan HK Agada CA Isola TO. bacterial load and antimicrobial profile of *escherichia coli* and *listeria spp*. isolates from muscle tissues of slaughtered cattle at a major abattoir in Ibadan, South-Western Nigeria. J Basic Appl Sci. (2014) 10:299–305. 10.6000/1927-5129.2014.10.39

[29] Adzitey F. Prevalence of Escherichia coli and Salmonella spp. in beef samples sold at tamale metropolis, Ghana. Int J Meat Sci. (2015) 5:8–13. 10.3923/ijmeat.2015.8.13

[30] Iroha IR Ugbo EC Ilang DC Oji AE Ayogu TE. Bacteria contamination of raw meat sold in Abakaliki, Ebonyi State Nigeria. J Health Epidemiol. (2011) 3:49–53. Available online at: http://www.academicjournals.org/jphe

[31] Soltan Dallal MM Sharifi Yazdi MK Mirzaei N Kalantar E. Prevalence of *Salmonella* spp. in packed and unpacked red meat and chicken in South of Tehran. Jundishapur J Microbiol. (2014) 7:1–4. 10.5812/jjm.9254 25147699 PMC4138620

[32] National Institute for Communicable Diseases (NICD). Situation Report on Listeriosis Outbreak South Africa. (2017). Available online at: https://www.nhls.ac.za/situation-report-on-listeriosis-outbreak-south-africa-2017 (accessed February 12, 2018).

[33] Gómez D Iguácel L Rota M Carramiñana J Ariño A Yangüela J. Occurrence of *Listeria monocytogenes* in ready-to-eat meat products and meat processing plants in Spain. Foods. (2015) 4:271–82. 10.3390/foods4030271 28231204 PMC5224534

[34] Dubey JP Bhaiyat MI Allie C Macpherson CNL Sharma RN Sreekumar C. Isolation, tissue distribution, and molecular characterization of Toxoplasma gondii from chickens in Grenada, West Indies. J Parasitol. (2005) 91:557–60. 10.1645/GE-463R 16108546

[35] Hosein S Limon G Dadios N Guitian J Blake DP. *Toxoplasma gondii* detection in cattle: A slaughterhouse survey. Vet Parasitol. (2016) 228:126–9. 10.1016/j.vetpar.2016.09.001 27692313

[36] Amdouni Y Rjeibi MR Rouatbi M Amairia S Awadi S Gharbi M. Molecular detection of Toxoplasma gondii infection in slaughtered ruminants in Northwest Tunisia. Meat Sci. (2017) 133:180–4. 10.1016/j.meatsci.2017.07.004 28711018

[37] Eberhard ML Ortega YR Hanes DE Nace EK Do RQ Robl MG. Attempts to establish experimental *Cyclospora cayetanensis* infection in laboratory animals. J Parasitol. (2000) 6:577–82. 10.2307/3284875 10864257

[38] Chu DM Sherchand JB Cross JH Orlandi PA. Detection of *Cyclospora cayetanensis* in animal fecal isolates from Nepal using an FTA filter-base polymerase chain reaction method. Am J Trop Med Hygiene. (2004) 71:373–9. 10.4269/ajtmh.2004.71.373 15516629

[39] Thurston-Enriquez JA Watt P Dowd SE Enriquez R Pepper IL Gerba CP. Detection of protozoan parasites and microsporidia in irrigation waters used for crop production. J Food Prod. (2002) 65:378–82. 10.4315/0362-028X-65.2.378 11848571

[40] FAO. Designing and Implementing Livestock Value Chain Studies – A Practical Aid for Highly Pathogenic and Emerging Disease (HPED) Control. Rome: FAO Animal Production and Health Guidelines. (2012).

[41] Scoones I. Avian Influenza: Science, Policy and Politics. Pathways to Sustainability Series. London, Washington, DC: Earthscan. (2010).

